# Comparison of 2 ways of performing ristocetin-induced platelet agglutination mixing study for diagnosis of type 2B von Willebrand disease. Response to the publication of Soleimani et al

**DOI:** 10.1016/j.rpth.2023.100286

**Published:** 2023-07-01

**Authors:** Emmanuel J. Favaloro, Maha Othman

**Affiliations:** 1 Haematology, Sydney Centres for Thrombosis and Haemostasis, Institute of Clinical Pathology and Medical Research, NSW Health Pathology, Westmead Hospital, Westmead, New South Wales, Australia; 2 School of Dentistry and Medical Sciences, Faculty of Science and Health, Charles Sturt University, Wagga Wagga, New South Wales, Australia; 3 School of Medical Sciences, Faculty of Medicine and Health, University of Sydney, Westmead Hospital, Westmead, New South Wales, Australia; 4 Department of Biomedical and Molecular Sciences, Queen’s University, Kingston, Ontario, Canada; 5 School of Baccalaureate Nursing, St. Lawrence College, Kingston, Ontario, Canada; 6 Clinical Pathology Department, School of Medicine, Mansoura University, Egypt

To the Editors,

We are thankful for the opportunity to respond to the recent letter from Soleimani et al. [1] in response to a prior publication of ours in this journal [2]. The authors provided further evidence of the utility of ristocetin-induced platelet agglutination/aggregation (RIPA) and RIPA mixing studies to help differentially identify type 2B von Willebrand disease (VWD) vs platelet type (PT) VWD. In brief, the first step requires identifying whether RIPA testing shows low-dose ristocetin responsiveness (typically between 0.5 and 0.7 mg/mL), which indicates either 2B VWD (due to gain-of-function von Willebrand factor [VWF]) or PT VWD (due to gain-of-function glycoprotein Ib [GPIb]). The next step is to differentiate between the 2, given different management pathways, which requires either genetic testing of both *VWF* and *GPIb* or RIPA mixing studies to determine the origin of the defect that leads to the enhanced response (plasma = 2B VWD, platelets = PT VWD); then, if required, confirmation can be finalized by genetic testing of either *VWF* or *GPIb,* according to RIPA mixing findings. In their study, RIPA mixing identified a plasma origin and thus, 2B VWD, which was later confirmed genetically for 2 investigated cases [1]. Notably, 2 approaches to RIPA mixing were investigated, as previously detailed in a methodology article [3]. This recent confirmation provides further validation of RIPA mixing studies as a workable solution for identification/discrimination of 2B/PT VWD, especially given that the latest 2021 American Society of Hematology, International Society on Thrombosis and Haemostasis, National Hemophilia Foundation, and World Federation of Hemophilia guidelines on VWD diagnosis seem to favor genetic testing, which is not always available to laboratories or which may be cost prohibitive for laboratories in developing/resource-poor countries. We do not think we “challenged the use of RIPA mixing studies, outlining the lack of applicability in laboratories and challenging interpretation for laboratories and physicians” in our initial article [2], as they stated [1]; rather, we were trying to encourage further studies to validate this approach, which the authors thankfully provided. It is not surprising that they found the Argentinean method easier to perform than the Westmead method, with these independently developed by Frontroth and Favaloro, also noting the lower volume required with the Argentinean method, applicable to pediatric use and developed by Frontroth working in a pediatric hospital [3]. The Westmead method was instead developed in an adult hospital [3]. Perhaps also of interest is that the Westmead method was recently used in Australia to work up a patient with 2B VWD at the other side of the age spectrum, in which an initial, perpetuating, but incorrect diagnosis of idiopathic thrombocytopenia took a lifelong of 86 years to correct [4]. The patient had over 50 full blood counts recorded between 1999 and 2017, with platelet count ranging between 42 × 10^9^/L and 170 × 10^9^/L, perpetuating the myth of idiopathic thrombocytopenia. The correction to 2B VWD finally transpired after eventual referral to a hemostasis laboratory that applied RIPA/RIPA mixing to provisionally identify 2B VWD, later confirmed by genetic *VWF* testing that identified a previously undescribed VWF variant (c.4130C>G; p.Ala1377Gly).
